# Biofunctional roles of estrogen in coronavirus disease 2019: Beyond a steroid hormone

**DOI:** 10.3389/fphar.2022.1003469

**Published:** 2022-10-19

**Authors:** Zhong-Ping Wang, Mao Hua, Tai Jiu, Ri-Li Ge, Zhenzhong Bai

**Affiliations:** ^1^ Clinical Medicine, School of Medicine, Qinghai University, Xining, China; ^2^ Department of Respiratory and Critical Diseases, Affiliated Hospital of Qinghai University, Xining, China; ^3^ Research Center of High-Altitude Medicine, School of Medicine, Qinghai University, Xining, China; ^4^ Joint Lab of Qinghai-Utah for High Altitude Medicine, School of Medicine, Qinghai University, Xining, China

**Keywords:** COVID-19, SARS-CoV-2, estrogen, estrogen therapy, prognosis, epidemic, immune response

## Abstract

The coronavirus disease 2019 (COVID-19), caused by severe acute respiratory syndrome coronavirus 2 (SARS-CoV-2), epidemic poses a major global public health threat with more than one million daily new infections and hundreds of deaths. To combat this global pandemic, efficient prevention and management strategies are urgently needed. Together with the main characteristics of COVID-19, impaired coagulation with dysfunctions of the immune response in COVID-19 pathophysiology causes high mortality and morbidity. From recent clinical observations, increased expression of specific types of estrogen appears to protect patients from SARS-CoV-2 infection, thereby, reducing mortality. COVID-19 severity is less common in women than in men, particularly in menopausal women. Furthermore, estrogen levels are negatively correlated with COVID-19 severity and mortality. These findings suggest that estrogen plays a protective role in the pathophysiology of COVID-19. In this review, we discuss the potential roles of estrogen in blocking the SARS-CoV-2 from invading alveolar cells and replicating, and summarize the potential mechanisms of anti-inflammation, immune modulation, reactive oxygen species resistance, anti-thrombosis, vascular dilation, and vascular endothelium protection. Finally, the potential therapeutic effects of estrogen against COVID-19 are reviewed. This review provides insights into the role of estrogen and its use as a potential strategy to reduce the mortality associated with COVID-19, and possibly other viral infections and discusses the possible challenges and pertinent questions.

## Introduction

In December 2019, an outbreak of coronavirus disease (COVID-19) caused by severe acute respiratory syndrome coronavirus 2 (SARS-COV-2) was reported in Wuhan, China (Guan et al., 2020; Wang et al., 2020), and has since rapidly spread globally (Young et al., 2020). As of 30 September 2022, over 652 million cases have been reported worldwide with a death toll of more than 6.5 million (WHO, 2022a). The huge spread of COVID-19 has created enormous challenges for global healthcare systems and economies. SARS-CoV-2 is a single-stranded RNA virus surrounded by a capsule (Han et al., 2020). Similar to the Middle East respiratory syndrome coronavirus and severe acute respiratory syndrome coronavirus 1 (SARS-CoV-1), SARS-CoV-2 also begins as an infection of the upper respiratory tract and over time invades the lower respiratory tract, eventually leading to fatal pneumonia (Wu et al., 2021).

**FIGURE 1:**
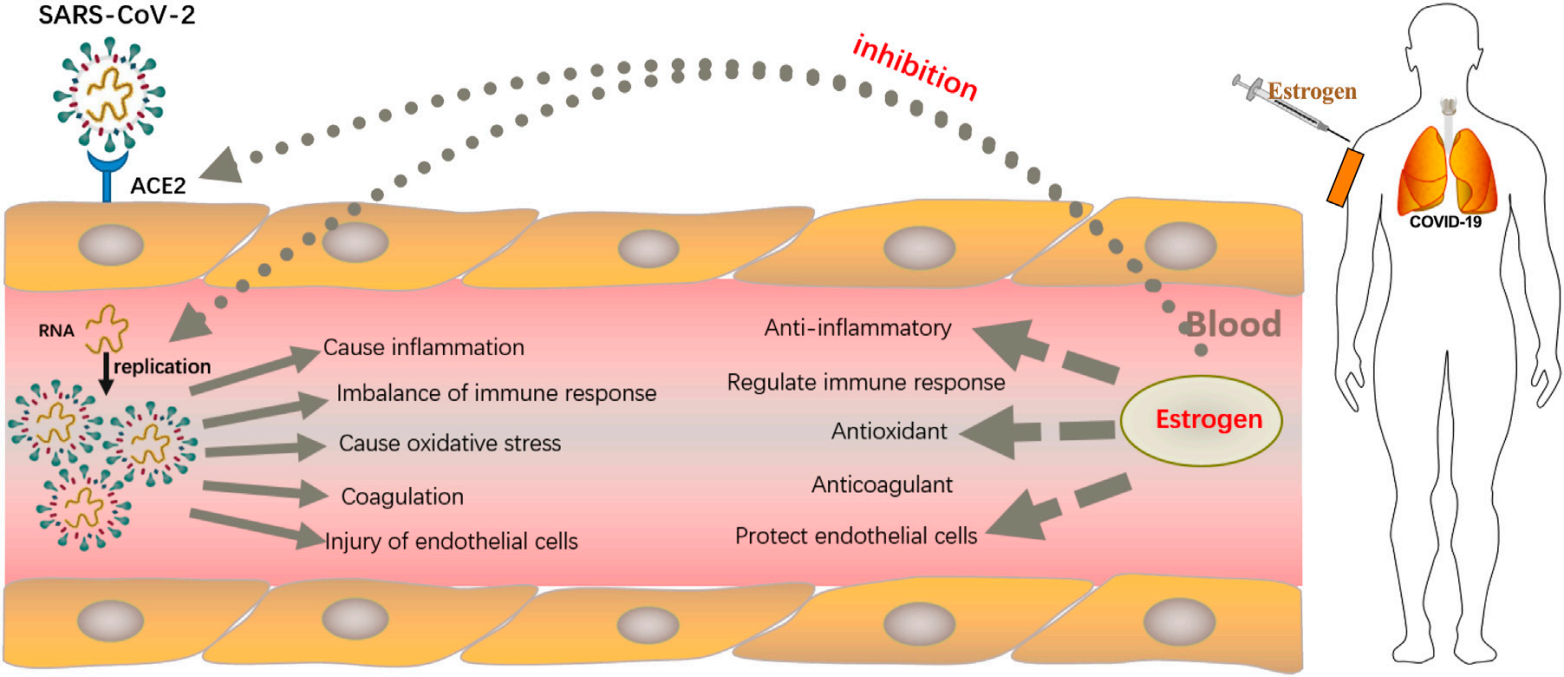
Strategies employed by estrogen in resisting SARS-CoV-2 infection. After binding to the angiotensin-converting enzyme 2 (ACE2) receptor on host cells through the spike protein, which lyses and fuses the cell membrane into host type 2 alveolar epithelial cells, SARS-CoV-2 enters the host cell and begins to replicate to produce more viral nucleocapsids, which requires the participation of the main viral protease enzyme. Thereafter, proliferated SARS-CoV-2 causes damage to alveolar macrophages, the tissue microenvironment releases proinflammatory cytokines and chemokines that promote the attraction of macrophages, neutrophils, and T cells, which can lead to uncontrolled inflammation, immune disorders, oxidative stress, damage to endothelial cells, and fatal clots. Estrogen, mostly administered subcutaneously, not only reduces the expression of ACE2 receptors on host type 2 alveolar epithelial cells, thereby, preventing the entry of SARS-COV-2 virus into host cells, but also inhibits viral replication by occupying the active site of the protease required for viral replication. In addition, estrogen has anti-inflammatory, antioxidant, immune cell regulatory, antithrombotic, and endothelial cell protection properties.

SARS-CoV-1 and SARS-CoV-2 share 79.6% homology (Zhou et al., 2020; Stukalov et al., 2021). During the previous SARS-CoV-1 and Middle East respiratory syndrome coronavirus epidemics, estrogen and its analogs were reported to play a protective role against SARS coronavirus infections (Channappanavar et al., 2017; Islam et al., 2021). In addition, animal studies have shown that reducing estradiol or using estrogen receptor antagonists is conducive to severe adult respiratory syndrome coronavirus infection (Channappanavar et al., 2017; Suba, 2020). Throughout the SARS-CoV-2 pandemic, the rate of hospitalization for men remains much higher than that for women (Gomez et al., 2021; Mohamed et al., 2021). Moreover, men have more severe symptoms and higher mortality (Alwani et al., 2021; Ferretti et al., 2022). A recent retrospective study of estrogen therapy in female patients with COVID-19 showed that those over 50 years of age receiving estrogen therapy had a 50% reduction in the risk of death from COVID-19 than those under 35 years of age (Seeland et al., 2020). An *in vitro* study showed that the viral load of cell infection decreased after treating SARS-CoV-2 infected cells with estrogen (Lemes et al., 2021). New studies have shown that estrogen induces a stronger response to the vaccine (Heriyanto et al., 2021; Uwamino et al., 2022). Moreover, plant flavonoids, known as phytoestrogens, surprisingly affect multiple processes including viral penetration into the host cells, replication of viral nucleic acids, and release of viral particles from cells infected with SARS-CoV-2 (Rahman et al., 2022). These findings suggest the potential protective role of estrogen in patients with COVID-19 (Figure 1). However, the exact role of estrogen in COVID-19 and the associated underlying mechanisms remain nebulous. Here, we summarize the potential roles of estrogen in interfering with the invasion and replication of SARS-CoV-2 virus and discuss the underlying mechanisms as well as the challenges in using estrogen to curtail this global pandemic. Briefly, we have organized this mini review as follows: 1) Overview of estrogen; 2) Estrogen and COVID-19; 3) Deficiencies in estrogen therapy and clinical treatment challenges; and 4) Conclusion and future directions.

## Overview of estrogen

### Resources and classification

Estrogen is a steroid hormone with lipophilic properties and plays an important role in the growth and development of several organ systems, including the breast, uterus, neuroendocrine, skeletal, and cardiovascular systems (Hamilton et al., 2017). Most of the estrogen in the body is originated from the ovaries, luteum, and placenta of premenopausal women, whereas a small portion comes from non-ovarian organs, such as the adrenal gland, liver, heart, skin, brain, male testicles, and adipose tissue (Barakat et al., 2016; Catenaccio et al., 2016).

Three types of estrogens have been identified in the human body: estrone (E1), 17β-estradiol (E2), and estriol (E3) (Cui et al., 2013), among which E2 has the strongest biological activity (Medzikovic et al., 2019) and is the main and most effective form of estrogen in female blood circulation (Rettberg et al., 2014). E1 is synthesized by the adrenal dehydroepiandrosterone in the adipose tissue and is more predominant after menopause; E2 is the main product of the biosynthetic process and the most effective estrogen before menopause; E3, produced from E1 and formed by 16A hydroxylation, is the weakest, and yet it plays an important role during pregnancy (Lee et al., 2019). E1 is converted into E2 by the action of 17β hydroxylated steroid dehydrogenase 1 (17β-HSD1). Conversely, 17 β-hydroxylated steroid dehydrogenase 2 (17β-HSD2) converts E2 back to E1. This process is reversible; however, the conversion rate from E1 to E2 is slow. E2 also produces different metabolites *via* different cytochrome P450 enzymes and has been shown to play different biological roles in lung disease (Hood et al., 2016; Docherty et al., 2019).

### Interaction of estrogen with cellular receptors

Estrogen can easily pass through the cell membrane and bind to estrogen receptors (ERs), which mediate its physiological effects. Hence, ER is the primary target of the regulatory function of estrogen and affects diseases of multiple organ systems, such as the cardiovascular and skeletal systems (Luo and Kim, 2016).

Most estrogen receptors are classified as ligand-activated transcription factors in the steroid family. To date, two classical ERs, namely nuclear estrogen receptor (nER) and membrane estrogen receptor (mER), have been identified (Fuentes and Silveyra, 2019). There are two subtypes of nERs: ERα and ERβ (Dama et al., 2021). Erα, discovered in 1958 by Elwood Jenson, is diffusely scattered in the testicles, uterus, ovaries, prostate, skeletal muscle, kidney, skin, and at other sites (Russell et al., 2019). ERβ, isolated in 1996 by Kuiper et al. (1996), is widely distributed in the ovary, colon, kidney, brain tissue, as well as in the male reproductive system. Through the regulatory effect of different genes, ERα and ERβ have different effects on the disease state. Interestingly, ERβ not only exerts a protective effect against cardiopulmonary diseases, but also has antifibrotic (Pedram et al., 2010), antihypertrophic (Hajializadeh and Khaksari, 2022), anti-inflammatory (Umar et al., 2011), and vasodilation properties (Mishra et al., 2019). ERβ promotes the production of various angiogenic factors to regulate angiogenic NO and exerts vascular dilatation effects (Iorga et al., 2017). Estrogen can reduce the pressure caused by pulmonary vessels in the pathological state to protect the pulmonary arteries (Lahm et al., 2012). There is evidence that some target cells can respond to estrogen without the involvement of an ER (Thomas & Gustafsson, 2011), which is attributed to the G-protein-coupled estrogen receptors (GPERs) containing G-protein-coupled receptor 30 (GPR30) and ER-X that mediate fast mER-associated estrogen effects (Knowlton and Lee, 2012; Tang et al., 2019). GPR30 is expressed in the adrenal medulla, hippocampus, renal pelvis, cortex, hypothalamus, and ovaries (Xiang et al., 2019). The expression of ER-X is tightly regulated during the developmental stages of the brain, uterus, and fetus. In adults, ER-X is expressed at low levels in normal physiological states, but its expression increases following ischemic injury (Kiyama and Wada-Kiyama, 2015).

### Biosynthesis and metabolism of estrogen

**FIGURE 2:**
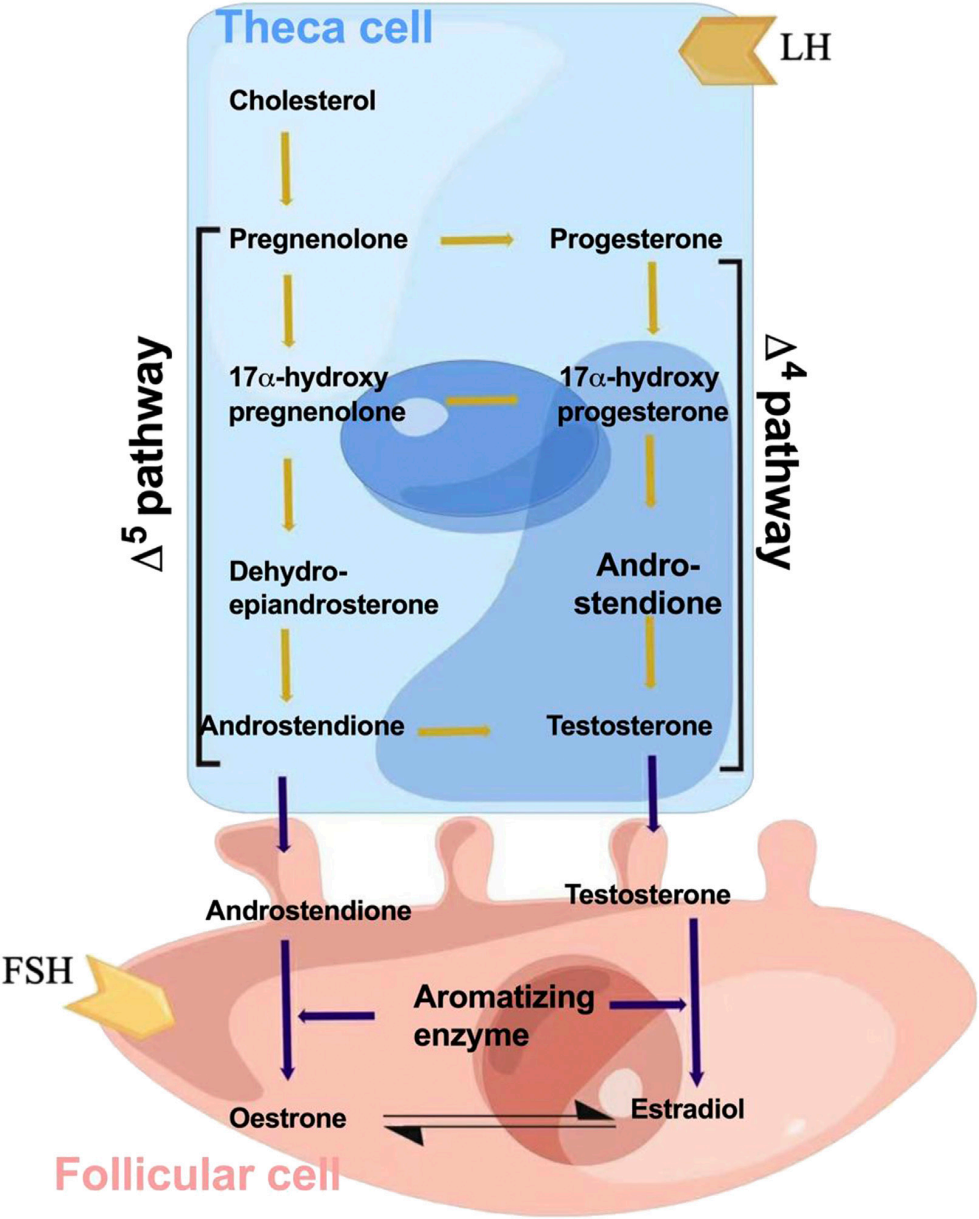
Schematic for estrogen synthesis in the ovaries. The dual-cell theory or two-gonadotropin theory of estrogen synthesis explains that estrogen is produced from endometrial and granulosa cells in the follicles through the synergistic action of follicle-stimulating hormone (FSH) and luteinizing hormone (LH). The endometrial cells of follicles use cholesterol as a raw material under the action of the luteinizing hormone and convert it into pregnenolone *via* cytochrome P450 enzymes. Thereafter, pregnenolone is converted into progesterone through the action of 3β-hydroxysteroid dehydrogenase. Progesterone is then oxidized to dehydroepiandrosterone and androstenedione by 17α-hydroxylase. Androstenedione is converted to testosterone by 17β-hydroxysteroid dehydrogenase type 3 (HSD17B3). The resulting androstenedione and testosterone then diffuse into the granulosa cells through the basement membrane. Aromatase activity is enhanced by FSH, and androstenedione is then converted to estrone and testosterone is converted to estradiol as the end products.

Estrogen is produced by endometrial and granulosa cells in the follicles through the synergistic action of follicle-stimulating hormone (FSH) and luteinizing hormone (LH) (Barakat et al., 2016). This is referred to as the two-cell two-gonadotropin theory of estrogen synthesis, also known as the dual-cell theory (Figure 2). First, the endometrial cells of follicles use cholesterol as a raw material under the action of LH and convert it into pregnenolone *via* cytochrome P450 enzymes (Bassi et al., 2021). Pregnenolone in turn is converted into progesterone through the action of 3β-hydroxysteroid dehydrogenase. Progesterone is then oxidized to dehydroepiandrosterone and androstenedione by 17α-hydroxylase (Manca et al., 2012). Androstenedione is converted to testosterone by 17β-hydroxysteroid dehydrogenase type 3 (HSD17B3), which is expressed almost exclusively in testicular interstitial cells (Yazawa et al., 2020). The resulting androstenedione and testosterone then diffuse into the granulosa cells through the basement membrane (Biegon et al., 2020). Aromatase activity is enhanced by FSH (Cooke et al., 2017). Androstenedione is then converted to estrone and testosterone is converted to estradiol (Fukami and Ogata, 2022). A small proportion of synthetic estrogen enters the follicular cavity, whereas a large proportion enters the bloodstream, thereby regulating the differentiation and growth of target cells, such as the endometrium and breast. Estrogen is metabolized *via* sulfation, which is a strategy to control its activity. By conjugating with sulfonic acid groups, sulfotransferase converts E1, E2, estrogen precursors, and metabolites (dehydroepiandrosterone and 2-Me) to Sulfated E1, E2, and 2-ME. These inactive sulfated steroids do not activate ERS, and being hydrophilic, they are more easily excreted by the kidneys (Sánchez-Guijo et al., 2016).

### Physiological roles of estrogen

Estrogen promotes the development of female secondary sexual characteristics and plays an important role in regulating the structure and function of the female reproductive system (Vasquez, 2018). Estrogen can also promote the development of the uterus; it relaxes the cervical opening during ovulation, promotes hypertrophy of the uterine smooth muscle and contraction of the fallopian tube and cilia swing, and makes vaginal secretions acidic to resist infection, besides being involved in follicular and external genital development in coordination with FSH (Chou and Chen, 2018). Estrogen also has important effects on other systems in the body. For instance, it can promote bone growth and development, thereby increasing the height of adolescent females (Iravani et al., 2017). Moreover, it improves lipid composition, prevents atherosclerosis, and protects the cardiovascular system (Knowlton and Lee, 2012). It also promotes the growth, differentiation, and regeneration of neurons (Brann et al., 2022). High estrogen levels can cause fluid movement between tissues, leading to the retention of sodium and water (Mallareddy et al., 2007). In addition, estrogen plays an important role in the occurrence, development, and prognosis of COVID-19 through its anti-inflammatory effects (Pelekanou et al., 2016); regulation of immune response (Kovats, 2015); antioxidative (Tsialtas et al., 2021), antithrombotic (Lee and Syed, 2022), and vasodilatory properties; and by protecting endothelial cells (Diebel et al., 2021).

## Estrogen and COVID-19

### Estrogen inhibits the replication of SARS-CoV-2

SARS-CoV-2 consists of four structural proteins—the spike, membrane, envelope, and nucleocapsid proteins. Of these, the spike protein protrudes from the virus surface and plays an important role in its attachment to host cells and in the penetration of cell membranes (Ke et al., 2020). SARS-CoV-2 binds to the angiotensin-converting enzyme 2 (ACE2) receptor on host cells through the spike protein, which lyses and fuses the cell membrane with host type 2 alveolar epithelial cells (Chambers et al., 2020; Jackson et al., 2022). SARS-CoV-2 then enters the host cell and begins to replicate to produce more viral nucleocapsids, which requires the participation of the main viral protease enzyme (Sun et al., 2022).

ACE2 is widely expressed in human cells, including alveolar and small intestinal epithelial cells, vascular endothelial cells, and smooth muscle cells (Kočar et al., 2021). Estrogen can affect the function and activity of SARS-CoV-2 (Mateus et al., 2022). It can reduce the expression of ACE2 receptors on host type 2 alveolar epithelial cells, downregulate the reactivity and number of ACE2 receptors (Seeland et al., 2020), reduce the binding of SARS-CoV-2 to ACE2 receptors, and prevent the entry of SARS-CoV-2 into host cells (Baristaite and Gurwitz, 2022). Furthermore, estrogen strongly interacts with the active site of the main protease required for viral replication, as a competitive inhibitor, thereby preventing SARS-COV-2 replication in host cells by occupying the active site of the protease enzyme (Mansouri et al., 2022). In addition, estrogen mitigates common mechanisms of injury by reducing the expression of ACE2 receptor and excessive inflammation, protecting endothelial and mitochondrial functions, insulin sensitivity, clotting cascade, immune response, cerebrovascular flow, and cognition (Saavedra, 2021). These properties of estrogen have already been employed in the treatment of inflammation, age-related changes, neurodegeneration, and metabolic disorders in many organs, including the brain and lungs (Saavedra, 2021).

### Immunoresistance and immunomodulation of estrogen against SARS-CoV-2

SARS-COV-2 induces apoptotic cell death in infected alveolar epithelial cells, which in turn causes the leakage of blood vessels in alveolar cells, initially triggering local inflammation, and subsequently the recruitment of circulatory immune cells into the infected lungs to confront the extracellular enveloped virus and ultimately destroy the virus-infected cells (Cao, 2020; Shi et al., 2020). Virus-laden lung cells also stimulate surrounding inflammatory cells (e.g., macrophages, mast cells, and dendritic cells), thereby producing many inflammatory mediators, including interleukins (IL) -12, -8, -6, -120, and -1, along with tumor necrosis factor-alpha (TNF-α), macrophage inflammatory protein 1α, interferon-β, monocyte chemotaxis protein, and interferon-γ (Qin et al., 2020). The release of a large number of inflammatory mediators activates the vascular and leukocyte responses of the host, resulting in the recruitment and infiltration of several leukocytes, which further exacerbates the inflammatory response. An excessive inflammatory response results in damage to the lung tissue, diffuse alveolar damage, and alveolar hemorrhage (Ackermann et al., 2020; Pernazza et al., 2020), leading to acute respiratory distress syndrome (Xu et al., 2020).

In addition, the effect of SARS-CoV-2 on the mechanism of cellular stress activation in immunoactive cells is important. SARS-CoV-2 induces polyclonal activation and lymphocyte apoptosis, pathological activation of macrophages, and immunosuppression (Xiong et al., 2020), resulting in a sharp reduction in the number of lymphocytes (mainly T cells) and cytokine storms. Lymphocytes are white blood cells that play a key role in adaptive immunity, enabling the immune system to recognize and remember antigens; they are the core components of the immune system that mainly comprise B and T lymphocytes. The B lymphocytes participate in humoral immunity and secrete antibodies. The T lymphocytes are involved in cellular immunity in the body. Cytokine storms can trigger an inflammatory environment, closely associated with severe tissue damage (Huang et al., 2020). Acute lymphocyte depletion and cytokine storm-induced excessive immune response can cause dysregulation of the immune response in patients with SARS-CoV-2 infection and can ultimately lead to septic shock, acute respiratory distress syndrome, and/or multiple organ failure, thereby increasing mortality (Jamal et al., 2021).

Estrogen is an important factor in inhibiting inflammation and in regulating immunity in COVID-19 (Arnold et al., 2022; Li et al., 2022). It is involved in regulating the development of inflammation and immune responses (Aksoy et al., 2014; Kowsar et al., 2014). The expression of proinflammatory and anti-inflammatory cytokines correlates with estrogen levels—low estrogen levels contribute to the production and regulation of proinflammatory cytokines, whereas high estrogen levels fight inflammation by inhibiting the production of inflammatory cytokines, such as IL-6, IL-1β, and TNF-α (Aksoy et al., 2014; Wang et al., 2021). Estrogen also reduces the risk of COVID-19 by downregulating inflammatory markers, such as chemokines and cell adhesion molecules (Pelekanou et al., 2016).

### The role of anti-reactive oxygen species in COVID-19

The human body constantly generates oxygen free radicals during normal metabolism, of which approximately 95% are reactive oxygen species (ROS), including superoxide anions (O_2_-), hydrogen peroxide (H_2_O_2_), hydroxyl free radicals (-OH), and peroxynitrite (ONOO-) (Galaris et al., 2019). Under optimum conditions, the body’s oxidative and antioxidant defense systems maintain a dynamic balance. Pathological damage occurs when there is an imbalance between antioxidant and oxidative effects, which is known as oxidative stress (OS) (Pignatelli et al., 2018). Generally, the excessive production of ROS and the imbalance of antioxidant mechanisms are critical to viral invasion and replication (Khomich et al., 2018). Infections with SARS-CoV-2 induce OS by producing large quantities of ROS, which is the primary cause of severe local and systemic tissue damage in COVID-19. Excessive ROS can increase the formation of neutrophil extracellular traps and inhibit the protective effects of the human immune system, that is, killing T cells involved in cellular immunity and preventing the specific immune response to SARS-CoV-2 from taking effect (Schönrich et al., 2020). Elevated levels of OS and reduced antioxidant capacity are associated with increased disease severity in hospitalized COVID-19 patients. Therefore, the use of antioxidants could be an effective treatment strategy for COVID-19 (Karkhanei et al., 2021).

Estrogen exerts an antioxidant effect on COVID-19 patients. In an animal study, rats with low estrogen levels had higher OS than those with high estrogen levels (Barp et al., 2002). Another *in vivo* study indicated that young women had lower biochemical markers of OS than their men counterparts (Kander, Cui, & Liu, 2017). In addition, clinical and experimental data indicate that women have stronger antioxidant capacity than that in men (Reckelhoff et al., 2019; Kim, 2022). Multiple studies have suggested that estrogen restrains vascular OS by regulating ROS production, thereby expediting the clearance of ROS and decreasing local ROS production (Arias-Loza et al., 2013). Other studies investigating the effect of estrogen on OS have shown that estrogen can reduce serum lipid peroxides and upregulate the body’s antioxidant status (Palmisano et al., 2018). Estrogen increases the levels of binding proteins produced by the liver, restores sodium balance, and distributes lipids by decreasing the levels of low-density lipoproteins and elevating the levels of high-density lipoproteins (HDL) (Ko and Kim, 2020). HDL levels have also been proven beneficial for COVID-19 prognosis (G. Wang et al., 2021).

### Estrogen as an anticoagulant

Abnormal coagulation is one of the prominent features of severe COVID-19. Severe endothelial injury and extensive thrombosis with microangiopathy have been observed in the lungs of COVID-19 patients (Ackermann et al., 2020) due to the extensive activation of the coagulation system in patients infected with SARS-COV-2, which increases the risk of blood clotting (Tang et al., 2020) and thrombosis. A large-scale cadaver lung autopsy in northern Italy revealed platelet-fibrin thrombosis in the small pulmonary arteries, suggesting that thrombosis, following SARS-COV-2 infection, plays a critical role in the death of COVID-19 patients (Carsana et al., 2020). Acute pulmonary embolism caused by venous thrombosis (Danzi et al., 2020; Zuckier et al., 2020) is life threatening in COVID-19 patients.

SARS-CoV-2 cells can stimulate surrounding inflammatory cells to release a large number of cytokines and chemokines to recruit many white blood cells (such as neutrophils and monocytes), which can enhance intravascular coagulation and thrombosis by activating the coagulation system and inhibiting the anticoagulant mechanism (Levi and van der Poll, 2017; Zuckier et al., 2020). Neutrophils infiltrate and activate host cells, producing a large number of toxic tissue mediators, such as ROS, to damage the host cells, and activate monocytes to express tissue factors (TF) that can stimulate the coagulation cascade and thrombin generation (Teuwen et al., 2020). Some cytokines, for instance, TNF-α and IL-6, are effective activators of TF-dependent coagulation cascades (Kerr et al., 2001). Moreover, anticoagulant pathways associated with endothelial cells, especially the protein C system, are also susceptible to damage by inflammatory cytokines such as IL-1β and TNF-α (Levi and van der Poll, 2017). Endothelial cells respond to the activation of IL-1β and TNF-α by expressing p-selectin, von Willebrand factor, and fibrinogen to promote coagulation, leading to the binding of a large number of platelets, which release VEGF, thereby enhancing the expression of TF in endothelial cells and further irritating the coagulation cascade (Teuwen et al., 2020). In other words, these events are conducive to increased fibrin production, which induces the deposition of blood clots in the microvascular system (Levi and van der Poll, 2017).

The effectiveness of estrogen as an anticoagulant depends on its administration. In a study on the effects of estrogen on postmenopausal women undergoing hysterectomy, those who received estrogen therapy alone had an increased risk of venous thromboembolism (VTE) compared with those in an oral placebo group (Anderson et al., 2004). However, some observational studies revealed that the risk of VTE in patients administered estrogen *via* subcutaneous injection was much lower than that in the orally administered estrogen group (Scarabin et al., 2003; Laliberté et al., 2018). The higher risk of VTE associated with oral estrogen is attributed to its effects on the liver, leading to an increase in some hepatogenic coagulation factors (Elger et al., 2017). The reason for the lower risk of VTE when estrogen is administered *via* the subcutaneous route is that estrogen can alter the number and function of platelets. Fox and Chambers (2006) observed significant reductions in platelet counts in ovariectomized mice treated with E2 (10 or 100 μg/kg per day, subcutaneously) for 10 days (Fox and Chambers, 2006). Valéra et al. (2012) also observed that the number of platelets in ovariectomized mice was reduced to varying degrees after treatment with E2 (200 μg/kg per day, subcutaneously) for 3 weeks (Valéra et al., 2012). An animal study showed that chronic subcutaneous injection of E2 reduced platelet reactivity and prolonged tail hemorrhage in mice (Valéra et al., 2017). Long-term high physiological levels of E2 in mice significantly reduced the reactivity of platelets *in vivo* and *in vitro* and the risk of thrombosis in mice (Valéra et al., 2015). E2 therapy has been shown to modulate the expression of platelet proteins, including β1 tubulin and several other proteins, which affect the production and activation of platelets. Estradiol can also inhibit platelet aggregation *in vitro* by promoting the excretion or reuptake of Ca^2+^ (Breithaupt-Faloppa et al., 2020).

Furthermore, estrogen reduces the expression of plasminogen activator inhibitor TYPE 1 and enhances the activity of tissue plasminogen through both non-receptor-mediated and receptor-mediated mechanisms of antioxidant activity, suggesting that estrogen has a beneficial effect on fibrinolysis (Yoon et al., 2020). Estradiol also protects endothelial function by activating endothelial nitric oxide (NO) synthase and increasing NO production (Park et al., 2021). Intact endothelial cells can maintain blood in blood vessels by inhibiting platelet adhesion and anticoagulant effects (Gu et al., 2017).

### Estrogen protects vascular endothelium and causes dilation of blood vessels

The vascular endothelium is an active paracrine, endocrine, and autocrine organ, essential for regulating vascular tone and maintaining vascular homeostasis (Flammer et al., 2012). Endothelial dysfunction is a major determinant of microvascular dysfunction; when the endothelial function is compromised, and vascular endothelial barrier function declines, the vascular permeability increases, and the endothelial cells secrete a variety of bioactive substances, which can cause vasoconstriction and subsequent ischemia of organs and tissue edema related to inflammation and blood coagulation (Joffre et al., 2020), causing further damage to the body.

SARS-CoV-2 infects the host by binding to ACE2 receptors expressed by alveolar epithelial cells, which are also widely expressed on vascular endothelial cells (Ferrario et al., 2005; Ackermann et al., 2020). A study of SARS-CoV-2 directly infecting engineered human blood vessels *in vitro* showed that SARS-CoV-2 could directly infect endothelial cells through ACE2 to alter vascular homeostasis (Monteil et al., 2020). After binding to the vascular endothelium, SARS-CoV-2 downregulated ACE2 receptor reactivity, and number of the receptor by affecting its expression in the vascular endothelium, which is conducive to the progression of pulmonary inflammation and profibrosis caused by local angiotensin II overactivity (Verdecchia et al., 2020). Reduced ACE2 expression can, in turn, indirectly activate the kallikrein—kinin system, ultimately leading to increased vascular permeability (Garvin et al., 2020; Teuwen et al., 2020). Virus-laden lung cells also stimulate the surrounding inflammatory cells to produce large amounts of cytokines, inducing an inflammatory response. Hypercytokinemia and the massive inflammatory response of the host cells can lead to endothelial dysfunction in COVID-19. For example, IL-6 can increase vascular permeability and promote the secretion of inflammatory cytokines by endothelial cells, thereby increasing the release of cytokines (Teuwen et al., 2020) and creating a vicious cycle. Electron microscopy of postmortem patient tissues further confirmed this conclusion, suggesting that SARS-CoV-2 can infect pulmonary endothelial cells and induce endodermatitis (Varga et al., 2020). Virus-infected vascular endothelium leads to extensive endothelial dysfunction associated with apoptosis through the immune-mediated recruitment of immune cells. In addition, the reduction in endothelial NO synthase activity, NO levels, and the release of VEGFs due to ARDS-induced systemic hypoxia have been deemed as pivotal pathogenic processes responsible for endothelial dysfunction after SARS-COV-2 infection (Martini, 2020).

Estrogen exerts a protective effect on endothelial function. Estrogen deficiency reduces NO content and causes an oxidative stress response to OS damage in endothelial cells. In a study on mice, Wassmann et al. (2002) used a selective estrogen receptor modulator, raloxifene, which increased the activity of endothelial NO synthase and the dependence of ER in reducing the release of ROS from blood vessel cells to improve endothelial dysfunction caused by high blood pressure; these vascular effects led to a significant reduction in blood pressure and vascular injury in male spontaneously hypertensive rats. Conversely, high physiological levels of estrogen increase the production of NO and reduce lipid peroxidation in ovariectomized rats (Yazğan et al., 2016). A study on estrogen therapy in healthy postmenopausal women showed that treatment with estrogen can significantly reduce catecholamine levels, mean blood pressure, and LDL levels, and increase HDL levels (Nii et al., 2016; Anagnostis et al., 2020). High HDL levels have also been shown to protect the vascular endothelium (Robert et al., 2021). Estrogen can activate the transcription of NO synthase by binding to the classical E2 receptor or GPER (Iorga et al., 2017), producing the vasodilator, NO, which spreads to vascular smooth muscle cells, activates guanylate cyclase, and increases the levels of cyclic guanosine phosphate (Förstermann et al., 2017). NO plays a direct role in tissue oxygen balance, organ perfusion, vascular remodeling, and metabolic demands by protecting endothelial cells and regulating vascular tension and diameter (Ally et al., 2020).

## Deficiencies in estrogen therapy and challenges in clinical treatment

**FIGURE 3:**
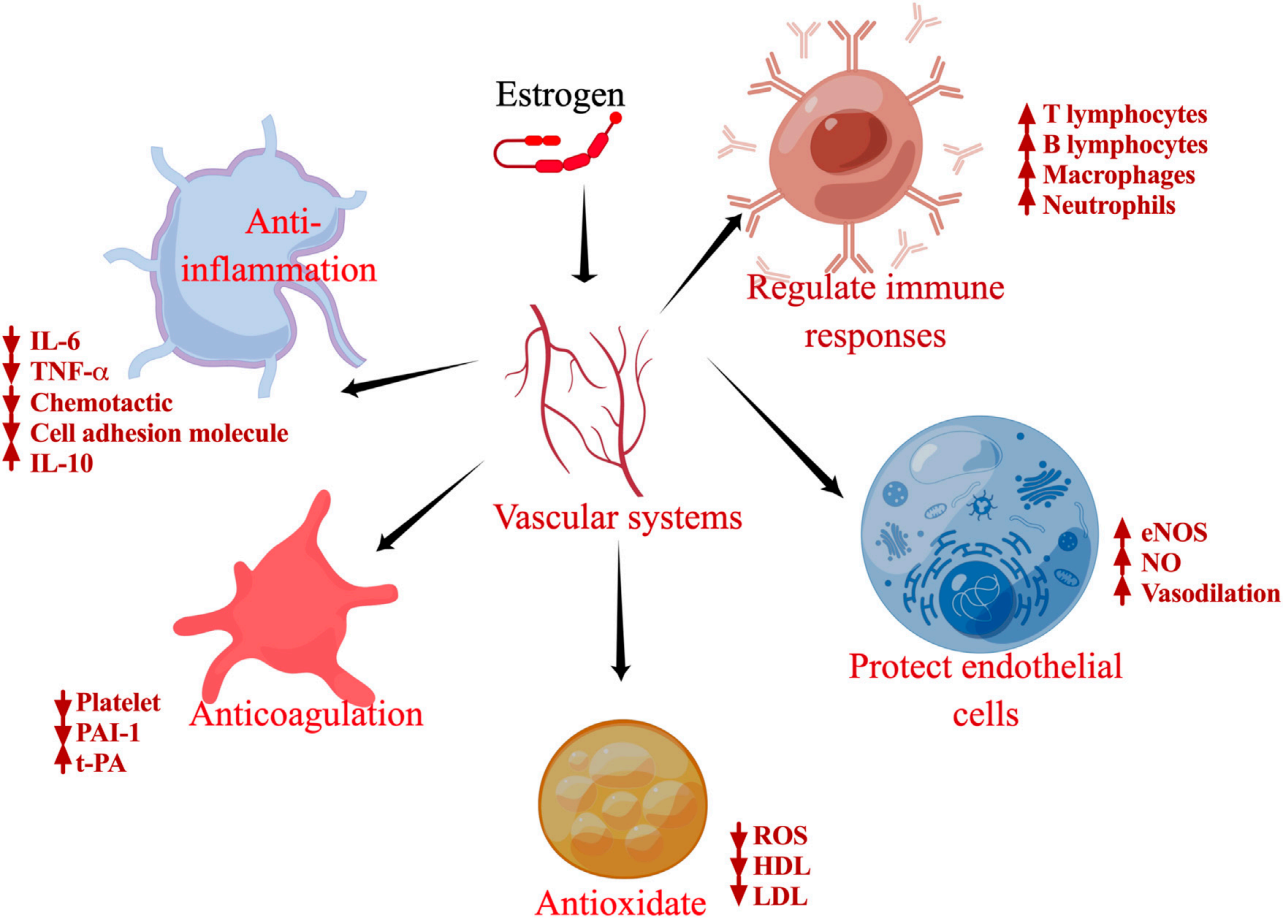
Schematic of the biological functions of estrogen. Specific effects of estrogen on inflammation, antioxidants, regulation of immune cells, antithrombosis, and protection of endothelial cells.

Owing to their numerous biological effects, as shown in Figure 3, estrogen and its analogs are associated with the treatment of a wide range of diseases, including neuroprotective therapies for ischemic stroke, Alzheimer’s disease, and Parkinson’s disease. Furthermore, estrogen remains the most effective treatment for symptomatic relief and for prevention of chronic disease in women with premature menopause due to primary ovarian insufficiency or medical etiology. Estrogen and its metabolites are also protective against pulmonary hypertension (Frump et al., 2021). Tamoxifen, the estrogen analog, has an inhibitory effect on Zika virus replication, which reduces Zika virus infection (Grady et al., 2021). Bristol et al. (2020) found that estrogen can downregulate the expression of early genes (e.g., E6 and E7) by inhibiting the human papillomavirus transcriptional long control region, ultimately attenuating the growth of human papillomavirus-positive epithelial cells (Bristo et al., 2020).

The applications of estrogen as an intervention strategy against COVID-19 are summarized in Table 1. Numerous studies have shown that transdermal estrogen therapy reduces the risk of death from COVID-19 in postmenopausal women (Seeland et al., 2020; Costeira et al., 2021; Sund et al., 2022), but for young premenopausal women, the risk of death from COVID-19 is the same regardless of estrogen therapy, probably because they have higher levels of endogenous estradiol (Seeland et al., 2020). A study on men treated with progesterone for COVID-19 showed that they required 3 days less supplemental oxygen and 2.5 days less hospital stay compared with a control group, suggesting a favorable effect of hormone therapy in men (Ghandehari et al., 2021). These findings may be applicable to the treatment of young female patients through personalized treatment strategy based on the level of estrogen in these patients, although the most efficacious estrogen dosage remains to be determined. Oral administration of estrogen is associated with a high risk of VTE, whereas subcutaneous therapy may have a slow onset and low bioavailability. The recently used nanoparticle (NP)-based drug delivery systems for the treatment of COVID-19 might provide a novel approach for estrogen therapy (Rahman et al., 2022; Rauf et al., 2022). However, the effective and efficient integration of estrogen into the NPS needs to be explored further. In addition, data on treatment duration and potential comorbidities are scarce, warranting further investigations.

**TABLE 1:** Estrogen interventions in the treatment of COVID-19.

Author and Year or Study id	Countries	Experimental Group (T)	Control Group (C)	People counting (T/C)	Proportion of women (%,T/C)	Mean age (T/C)	Main outcome measurement
Sund, et al. (2021)	Sweden	Estrogen reduction	Placebo	227/11,923	100/100	64.4/61.2	The absolute risk of death was 10.1% in the estrogen-reduced group and 4.6% in the control group
Sund et al. (2021)	Sweden	Estrogen increases	Placebo	2,535/11,923	100/100	60.9/61.2	The absolute risk of death was 2.1% in the estrogen-increased group and 4.6% in the control group
Ghandehari et al. (2021)	America	Progesterone therapy	Placebo	22/18	0/0	56 ± 17.3/54.6 ± 16	Compared with the control group, patients receiving progesterone required 3 fewer days of supplemental oxygen and 2.5 fewer days of hospital stay
Seeland et al. (2020)	Several countries	Progesterone therapy	Placebo	439/16,452	100/100	64.2/64.2	The death rate among women treated with estrogen was more than 50% lower than in the placebo group
Damb et al. (2021)	Britain	Progesterone therapy	Placebo	235/5,045	100/100	59	The absolute risk of death was more than four times higher in the control group than in the estrogen group
NCT04397718	America	Progesterone therapy	Placebo	132/66	—	—	The experiment was completed in June 2021, and the results were not published
NCT04801836	Several countries	Progesterone therapy	Placebo	81/81	—	—	The experiment was expected to be completed by November 2022
NCT04539626	Mexico	Progesterone therapy	Placebo	60	—	—	The experiment is expected to be completed by December 2022
CEP-UNIFESP 6864310320	Brazil	Progesterone therapy	DMSO	—	—	—	17β-Estradiol was able to significantly reduce (over 40%) cellular virus load (Animals with cell-lines)

## Conclusion and future directions

COVID-19 has a high morbidity and mortality, which poses a serious burden on healthcare systems and economic development worldwide. Therefore, strategies for the prevention and treatment of COVID-19 are urgently required. The pathophysiological features of COVID-19 include inflammatory storms, immune dysregulation, oxidative stress, thrombus formation in small pulmonary vessels, injury of pulmonary artery endothelial cells, and pulmonary artery spasm, which can lead to acute respiratory distress syndrome, liver dysfunction, multiple organ failure, and eventually, death.

To summarize, estrogen can inhibit the entry of virus particles, mitigate viral replication by reducing ACE2 receptor expression in the host cells, and curtail the activities of main viral proteases. Retrospective and clinical studies have shown that estrogen is associated with improved outcomes in patients with COVID-19 through the modulation of immune responses; anti-inflammatory, antioxidant, and antithrombotic effects; and protection of endothelial cells. Several studies have demonstrated that low estrogen levels are associated with higher morbidity and mortality, which indicates that high estrogen levels have beneficial effects against COVID-19. Pharmacological compounds that can increase the levels of estrogen, such as tamoxifen and progesterone therapy, are potential treatments for patients with COVID-19 that are already in preclinical studies. Notably, many studies related to other viral infections also support the notion that increasing estrogen levels in patients with viral infections may be a viable therapeutic option. However, increasing estrogen levels alone is not apparently enough to restore estrogen function. Therefore, further studies on the composition, structure, and function of estrogen in COVID-19 are required to fully understand the mechanism of estrogen action in COVID-19, and thereby, devise new therapeutic strategies against this disease.

## Abbreviations

COVID-19, coronavirus disease 2019; E2, 17β-Estradiol; E1, estrone; ERs, estrogen receptors; nER, nuclear estrogen receptor; mER, membrane estrogen receptor; ACE2, angiotensin-converting enzyme 2; TNF-α, tumor necrosis factor-α; IL, interleukins; ROS, reactive oxygen species; OS, oxidative stress; VTE, venous thromboembolism; NO, nitric oxide; HDL, high-density lipoprotein.
